# Genetic Analysis of *PLA2G6* in 22 Indian Families with Infantile Neuroaxonal Dystrophy, Atypical Late-Onset Neuroaxonal Dystrophy and Dystonia Parkinsonism Complex

**DOI:** 10.1371/journal.pone.0155605

**Published:** 2016-05-19

**Authors:** Saketh Kapoor, Mohd Hussain Shah, Nivedita Singh, Mohammad Iqbal Rather, Vishwanath Bhat, Sindhura Gopinath, Parayil Sankaran Bindu, Arun B. Taly, Sanjib Sinha, Madhu Nagappa, Rose Dawn Bharath, Anita Mahadevan, Gayathri Narayanappa, Yasha T. Chickabasaviah, Arun Kumar

**Affiliations:** 1 Department of Molecular Reproduction, Development and Genetics, Indian Institute of Science, Bangalore, 560012, India; 2 Department of Biotechnology, R.V. College of Engineering, Bangalore, 560059, India; 3 Department of Neurology, National Institute of Mental Health and Neuro Sciences, Bangalore, 560029, India; 4 Department of Neuroimaging and Interventional Radiology, National Institute of Mental Health and Neuro Sciences, Bangalore, 560029, India; 5 Department of Neuropathology, National Institute of Mental Health and Neuro Sciences, Bangalore, 560029, India; Odense University Hospital, DENMARK

## Abstract

Mutations in *PLA2G6* were identified in patients with a spectrum of neurodegenerative conditions, such as infantile neuroaxonal dystrophy (INAD), atypical late-onset neuroaxonal dystrophy (ANAD) and dystonia parkinsonism complex (DPC). However, there is no report on the genetic analysis of families with members affected with INAD, ANAD and DPC from India. Therefore, the main aim of this study was to perform genetic analysis of 22 Indian families with INAD, ANAD and DPC. DNA sequence analysis of the entire coding region of *PLA2G6* identified 13 different mutations, including five novel ones (p.Leu224Pro, p.Asp283Asn, p.Arg329Cys, p.Leu491Phe, and p.Arg649His), in 12/22 (54.55%) families with INAD and ANAD. Interestingly, one patient with INAD was homozygous for two different mutations, p.Leu491Phe and p.Ala516Val, and thus harboured four mutant alleles. With these mutations, the total number of mutations in this gene reaches 129. The absence of mutations in 10/22 (45.45%) families suggests that the mutations could be in deep intronic or promoter regions of this gene or these families could have mutations in a yet to be identified gene. The present study increases the mutation landscape of *PLA2G6*. The present finding will be useful for genetic diagnosis, carrier detection and genetic counselling to families included in this study and other families with similar disease condition.

## Introduction

Classic infantile neuroaxonal dystrophy ([INAD], MIM #256600), also known as Seitelberger’s disease, is a rare autosomal recessive neurodegenerative disease with a typical age of onset within the first two years of life. It is characterized by rapid progression of psychomotor regression and hypotonia evolving into spasticity and visual impairment with nystagmus due to optic atrophy. The clinical manifestation begins between 6–24 months of age, and usually leads to death by 10 years. This type of the disease was designated as ‘classic INAD’ [1]. The pathological hallmarks used in INAD diagnosis are axonal swellings and spheroid bodies in pre-synaptic terminals in both the central and peripheral nervous systems, which can be detected in skin biopsy. It is one of the forms of neurodegeneration with brain iron accumulation (NBIA), which is a heterogeneous group of progressive complex motor disorders characterized by the presence of high brain iron, particularly within the basal ganglia. The axonal swellings that occur in INAD are also seen in NBIA and other overlapping diseases such as pantothenate kinase-associated neurodegeneration (PKAN; MIM#610217). Some individuals with INAD show accumulation of high brain iron similar to NBIA. In some cases, the disease appears late in childhood and progresses slowly, designated as ‘atypical’ neuroaxonal dystrophy (atypical NAD/ANAD, MIM #610217) [2]. T2-weighted magnetic resonance imaging (MRI) of INAD and ANAD patients typically shows cerebellar atrophy with increased signal in cerebellar cortex and, occasionally, hypointensity in the pallida and substantia nigra [2].

*PLA2G6* consists of 17 exons and encodes an 806 amino acid long protein with a lipase motif (residues 517–521) and seven ankyrin repeats (residues 151–382) [3]. It is expressed in the brain, spinal cord, lung, kidney, gut, pancreas etc (http://www.proteinatlas.org). PLA2G6 catalyses hydrolysis of the membrane phospholipids at the *sn*-2 position of glycerophospholipids, leading to the production of a free fatty acid, usually arachidonic acid, and a lysophospholipid. Both catabolites have profound effects on the maintenance of cell membrane homeostasis or serve as precursors for biologically active metabolites involved in cell signal transduction [3]. In 2006, Morgan et al. [4] and Khateeb et al. [5] have independently shown that mutations in *PLA2G6* (phospholipase A2 Group 6), encoding a calcium-independent group VIA phospholipase A2 enzyme (iPLA2-VIA), cause INAD and ANAD. Mutations in this gene were also found in DPC [5]. Subsequently, mutations in this gene have been reported in individuals with INAD, ANAD and DPC from several countries [4–30]. However, there is no report on the genetic analysis of *PLA2G6* in Indian families with INAD, ANAD and DPC. Here, we report on the genetic analysis of 22 Indian families with INAD, ANAD and DPC.

## Materials and Methods

### Families

**Fig 1:**
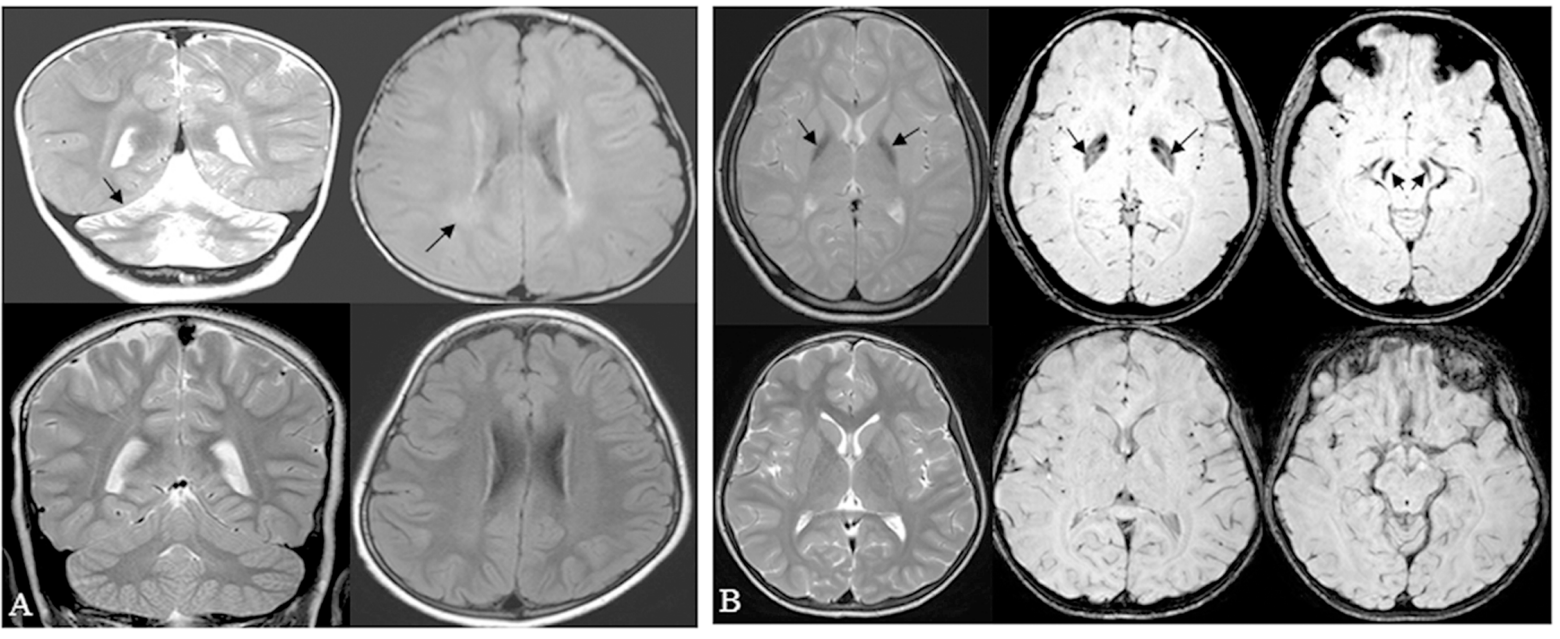
Magnetic resonance imaging findings. (A) Upper panel: Brain MRI of the 21-month old affected individual II-1 from family 2 with INAD showing cerebellar atrophy (arrow) on T2W coronal image (left side image), and bilateral peritrigonal hyperintensities (arrow) on FLAIR sequences (right side image). Lower panel: Compare with normal MRI sequences from a 2-year old boy. (B) Upper panel: Brain MRI of the 10-year affected individual II-1 from family 8 with ANAD showing bilateral global pallidus hypointensity (arrow) on T2W images (left side image) and blooming in globus pallidus and substantia nigra (arrow) on susceptibility weighted imaging (middle and right panels). Lower panel: Compare with normal MRI sequences from a 10-year old boy.

**Fig 2:**
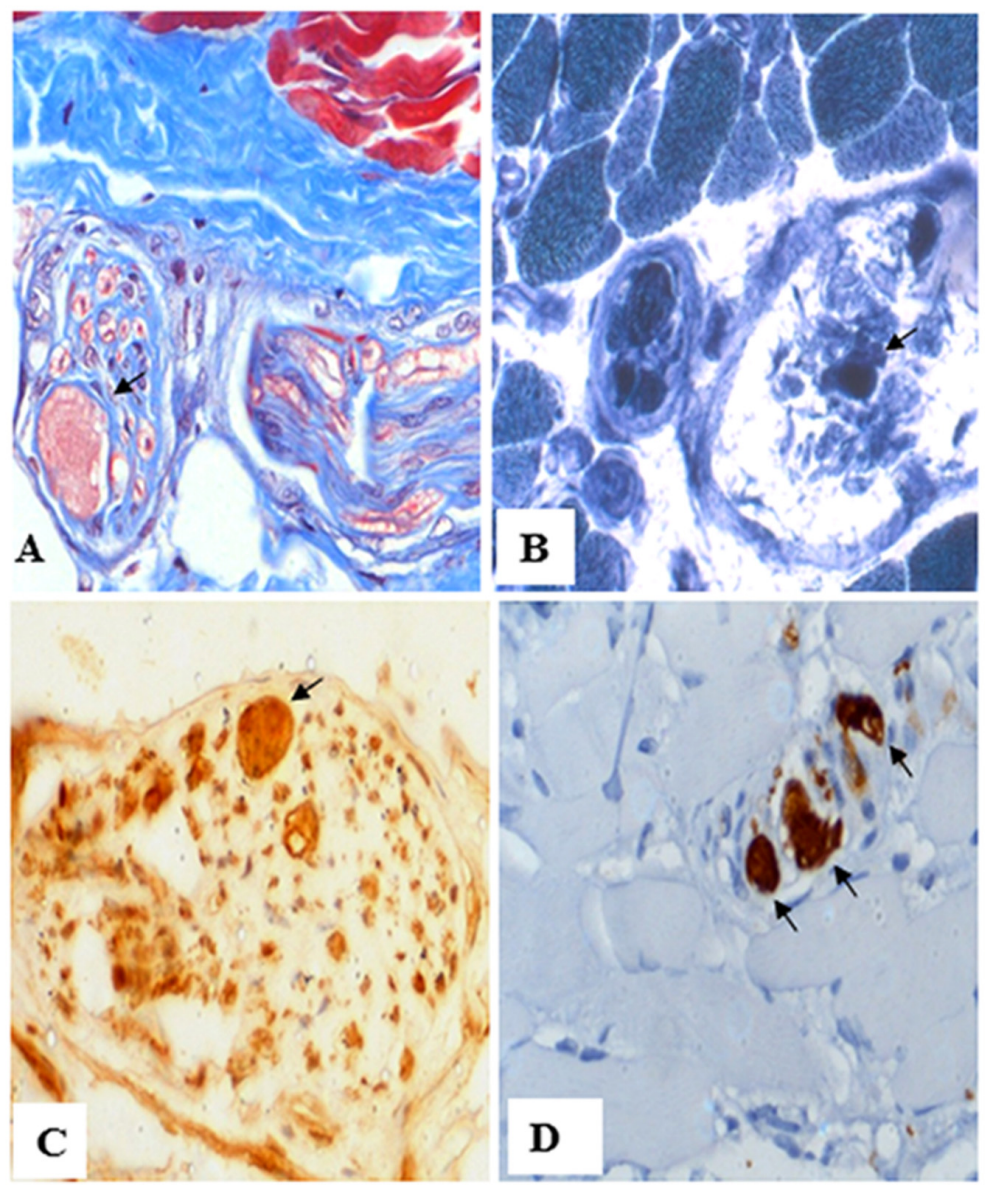
Muscle nerve pathology of affected individual II-1 from family 7. (A) Muscle biopsy reveals marked axonal distension of intramuscular nerve twig (arrow), using Masson's trichrome staining. (B) The intraaxonal contents in the distended axon contain NADH-TR (arrow). (C) Phosphorylated neurofilament staining (arrow). (D) Ubiquitin staining (arrow). Magnifications: (A) 200X; (B) 200X; (C) 400X; and, (D) 200X.

A total of 22 families (Fig A in S1 File) with INAD, ANAD and DPC were ascertained at the outpatient clinic of the Department of Neurology, National Institute of Mental Health and Neuro Sciences, Bangalore, Karnataka. Of 22 families, 13 showed clinical symptoms of INAD (family# 1, 2, 3, 5, 7, 9, 10, 11, 13, 15,16, 20 and 23), eight were diagnosed with ANAD (family# 4, 8, 12, 14, 18, 21, 24 and 25), and one (family# 22) had DPC. All patients with INAD had the classical clinical features characterized by neuroregression, ocular signs, optic atrophy, hypotonia, pyramidal signs and cerebellar atrophy. Magnetic resonance imaging also showed posterior periventricular white matter signal changes (Fig 1A). Patients with ANAD showed clinical features characterized by neuroregression and varying combinations of ataxia and extrapyramidal features, optic atrophy, cerebellar atrophy and hypointense globus pallidi (Fig 1B). Patients with DPC had dominant extrapyramidal features in the form of dystonia and parkinsonian features along with evidence of hypointense globus pallidi and cerebellar atrophy. A total of 15 patients underwent muscle biopsy. The muscle biopsies were snap frozen in chilled isopentane, cryosectioned and stained with hematoxylin-esoin and Masson's trichrome staining. Enzyme histochemical stains routinely used to study muscles biopsies included oxidative enzymes (NADH-TR that delineates sarcoplasmic reticulum and mitochondria) and ATPase stains (for fibre typing). Z serial sections were also subjected to immunohistochemistry by indirect immunoperoxidase method, using antibodies against phosphorylated neurofilament (monoclonal, 1:500 dilution) and ubiquitin (polyclonal, 1:100 dilution). Antibodies were purchased from Leica Biosystems (Chennai, India). The characteristic axonal spheroids on intramuscular nerve twigs were observed in five patients with INAD. An example is shown in Fig 2. The intramuscular nerve twigs within the muscle revealed distension of few axons (reaching 30–40 microns) forming "spheroids" (Fig 2A). Organelles and tubular proteins were reflected by accumulation of NADH-TR (Fig 2B). The distended axoplasm revealed dystrophic changes with contained abnormally phosphorylated neurofilament (Fig 2C) and ubiquitin (Fig 2D). All patients with dystonia symptoms were given a trial of levodopa in adequate dosages. Even though some patients showed mild improvement, the response was not satisfactory in most of them. Details of the clinical and imaging features of all the patients are given in Table 1.

**Table 1:** Clinical features of the affected individuals from 22 Indian families with INAD, ANAD and DPC.

SI.#	Family#	Diagnosis of the family	Affected individual	Sex/Age	Age of onset	Pyramidal signs	Optic atrophy	Nystagmus / Strabismus	Other signs	Axonal spheroids^a^	Cerebellar atrophy on MRI	Brain iron on MRI	White matter signal changes on MRI
1	1	INAD	II-1	F/5yr	21mo	+	+	+	Hypotonia	+	+	-	+
2	2	INAD	II-1	F/21mo	17mo	+	+	+	Hypotonia	+	+	+	+
3	3	INAD	II-1	F/3yr	18mo	+	+	+	Hypotonia	ND	+	-	+
4	4	ANAD	II-1	F/8yr	2yr	+	-	+	Dystonia	ND	+	++	-
5	4	ANAD	II-2	F/6yr	2yr	+	-	+	Dystonia	ND	-	++	-
6	5	INAD	II-2	M/22mo	21mo	+	+	+	Hypotonia	+	+	-	+
7	7	INAD	II-1	M/3yr	18mo	+	+	+	Hypotonia	+	+	-	+
8	8	ANAD	II-1	M/10yr	8yr	+	-	-	Dystonia, bradykinesia	-	+	++	-
9	9	INAD	II-1	F/3yr	18mo	-	+	+	Hypotonia, hyporeflexia	-	+	-	-
10	9	INAD	II-2	F/10mo	7mo	-	+	-	Hypotonia, hyporeflexia	ND	+	-	-
11	10	INAD	II-1	M/4yr	3yr	+	+	+	Hypotonia	+	+	-	+
12	11	INAD	II-1	F/6yr	8mo	+	+	+	Global development delay, neuroregression, hypotonia, dystonia	ND	+	-	-
13	12	ANAD	II-1	M/6yr	6yr	+	-	-	Dystonia	-	-	++	-
14	13	INAD	II-1	F/11mo	11mo	+	-	+	Dystonia	ND	+	-	-
15	14	ANAD	II-1	M/12yr	2yr	+	-	-	Developmental delay, early onset ataxia, slow saccades	ND	+	+	-
16	15	INAD	II-2	F/3yr	1yr	+	+	+	Hypotonia	-	+	-	-
17	16	INAD	II-1	F/3yr	9mo	-	-	-	Ataxia, dystonia, hypotonia, hyporelexia	-	+	+	-
18	18	ANAD	II-1	M/12yr	8yr	+	-	+	Ataxia, dystonia, choreiform movements	-	-	+	-
19	20	INAD	II-2	M/3yr	9mo	+	+	+	Hypotonia	-	+	-	+
20	21	ANAD	II-2	M/16yr	8yr	+	-	-	Ataxia, action myoclonus	-	-	++	+
21	22	DPC	II-1	M/37yr	31yr	+	-	-	Bradykinesia, rigidity	ND	+	++	-
22	23	INAD	II-2	M/4yr	1.5yr	+	+	+	Hypotonia	-	+	+	+
23	24	ANAD	II-1	M/16yr	9yr	+	-	-	Ataxia, dystonia	-	+	++	-
24	25	ANAD	II-1	F/11yr	8yr	+	-	-	Generalised dystonia	ND	+	++	-

INAD, infantile neuroaxonal dystrophy; ANAD, atypical neuroaxonal dystrophy; DPC, dystonia parkinsonism complex; F, female; M, male; mo, month; yr, year; +, symptom present; -, symptom absent; ND, not done.

^a^ Axonal spheroids detected in intramuscular nerve twigs on muscle biopsy.

### Genetic analysis

For genetic analysis, 3–5 ml of peripheral blood sample was drawn from each individual in a Vacutainer^TM^ EDTA tube (Becton Dickinson, Franklin Lakes, NJ) and used for genomic DNA isolation using a Wizard Genomic DNA extraction kit (Promega, Madison, WI). Participants provided their written informed consent to participate in this study, which was approved by the ethics committee of National Institute of Mental Health and Neuro Sciences. For mutation analysis, the entire coding region of *PLA2G6* (GenBank accession no. NM_003560.2), including intron-exon junctions, was amplified using 16 pairs of primers (Table A in S1 File). Primers were designed using the gene sequence from the UCSC Genome Bioinformatics site (http://www.genome.ucsc.edu). Sequence variants were identified by sequencing the PCR product from one affected individual from each family on an ABIprism A310-automated sequencer (Life Technologies, Carlsbad, CA). PCR was performed in a total volume of 25 μl containing 50 ng of genomic DNA, 1.5 mM MgCl_2_, 200 μM of each dNTP, 1X buffer and 1 unit of *Taq* DNA polymerase (Biotools, Bangalore, India), using an Eppendorf^TM^ Thermocycler. Before sequencing, PCR products were purified using a Gen Elute^TM^ gel extraction kit (Sigma-Aldrich, St. Louis, MO). Once a sequence variant was identified, all available first degree family members were sequenced to identify the variant. Allele-specific PCR (Table B in S1 File) or DNA sequencing was performed to determine if a specific variant was present in 100 ethnically matched normal controls.

To find the functional significance of changed amino acid residues, PLA2G6 protein sequences from different species were aligned by the ClustalW2 program (http://www.ebi.ac.uk/Tools/msa/clustalw2/). To predict the effect of mutations on PLA2G6 function, we used two bioinformatics programs: PolyPhen-2 (http://genetics.bwh.harvard.edu/pph2/) and Mutation Taster (http://www.mutationtaster.org). The output score from the PolyPhen-2 program ranges from 0 to a positive number, where 0 is neutral, and a high positive number is damaging to protein function. The output from the Mutation Taster program is a p (probability) value. A p value close to 1 indicates that the mutation is damaging to protein function.

## Results and Discussion

**Fig 3:**
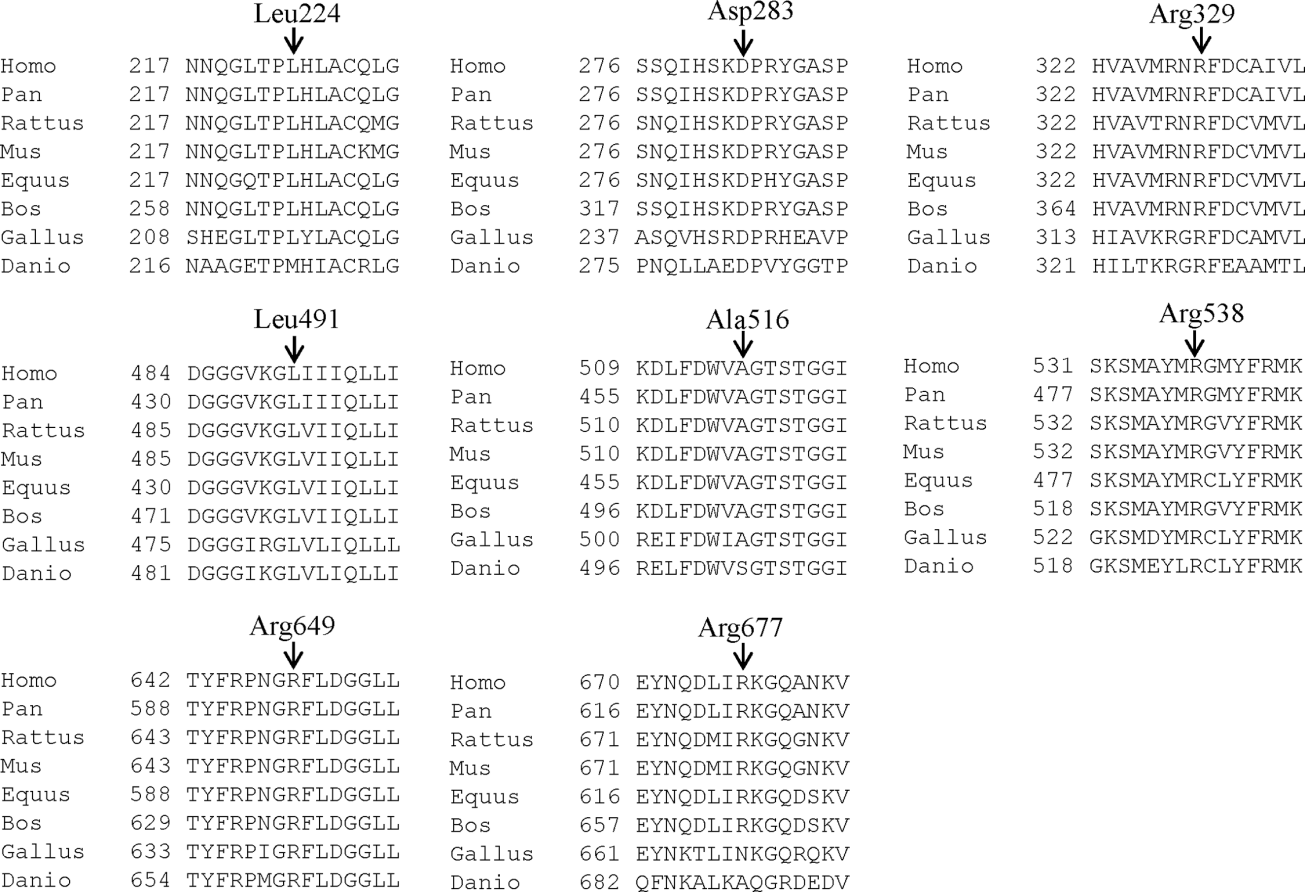
Conservation of amino acid residues across different species in PLA2G6. Arrows mark the conservation of amino acid residues Leu224, Asp283, Arg329, Leu491, Ala516, Arg538, Arg649 and Arg677 across different species. The number refers to the position of amino acid residue.

DNA sequence analysis of the entire coding region, including intron-exon junctions, of *PLA2G6* identified five known mutations (viz., p.Arg70*, p.Ala80Thr, p.Gly347Arg, p.Gly638Arg and p.Arg741Trp) (Table B and Table C in S1 File) and eight novel sequence variants (viz., p.Leu224Pro, p.Asp283Asn, p.Arg329Cys, p.Leu491Phe, p.Ala516Val, p.Arg538His, p.Arg649His and p.Arg677Leu) (Figs B-D in S1 File) in 12/22 (54.55%) families. Based on the following criteria, we considered the eight novel sequence variants as mutations. 1) The changes were segregating in the family. 2) The changed amino acids were highly conserved across species (Fig 3). 3) The changes were not observed in 100 normal controls (data not shown). 4) The PolyPhen-2 program predicted all eight changes to be probably damaging (Table 2). 5) The Mutation Taster program predicted all eight changes to be disease causing (Table 2). 6) All eight novel variants were absent in the 1000 Genomes database (http://www.1000genomes.org/). 7) Except p.Ala516Val (rs151108668), p.Arg538His (rs535486098) and p.Arg677Leu (rs369038599), all other five missense novel variants were absent in the ExAC (Exome Aggregation Consortium; http://exac.broadinstitute.org/) database, which contains whole-exome sequencing (WES) data of 60,706 unrelated individuals. The three variants, p.Ala516Val, p.Arg538His and p.Arg677Leu were found in the ExAC database in a heterozygous state only with the frequency of 1/20,836, 1/120,738 and 13/109,150 alleles, respectively. Of these, p.Ala516Val and p.Arg538His were found in a homozygous state in affected individuals of family 20 and 23, respectively (Table 2). Whereas p.Arg677Leu present in the affected individual II-1 of family 13 was found in a heterozygous state (Table 2). We were not successful in identifying the second mutation in this family. Taken together, 5/13 mutations (viz., p.Leu224Pro, p.Asp283Asn, p.Arg329Cys, p.Leu491Phe and p.Arg649His) identified are novel. The C scores ([31]; http://cadd.gs.washington.edu/), which highly rank pathogenic variants within individual genomes, ranges from 9.93 to 36 (Table 2). Further, all the 13 variants were found to be “pathogenic”, using the guidelines developed by the American College of Medical Genetics and Genomics (ACMG) for the interpretation of sequence variants [32].

**Table 2:** Details of the *PLA2G6* mutations identified during the present study.

SI. #	Family #	Diagnosis of the family	Mutation	Polyphen-2 score	Mutation Taster score	C score	Zygosity in the affected individual	Novel/known	ExAC database	Reference
1	1	INAD	c.2221C>T (p.Arg741Trp)	Probably damaging with a score of 1	Disease causing with a p value of 0.88	21.9	Homozygous	Known	Homozygous individual not known (1/17,550 alleles; only heterozygous individual known)	1,4,9,30
2	2	INAD	c.671T>C (p.Leu224Pro)	Probably damaging with a score of 1	Disease causing with a p value of 0.99	31	Heterozygous	Novel	Not present	Present study
3	3	INAD	c.1039G>A (p.Gly347Arg)	Probably damaging with a score of 1	Disease causing with a p values of 0.99	29.4	Homozygous	Known	Not present	1,4
4	4	ANAD	ND							
5	5	INAD	c.208C>T (p.Arg70*)	Probably damaging with a score of 1	Disease causing with a p value of 1	35	Homozygous	Known	Not present	11
6	7	INAD	c.985C>T (p.Arg329Cys)	Probably damaging with a score of 1	Disease causing with a p value of 0.99	36	Homozygous	Novel	Not present	Present study
7	8	ANAD	c.238G>A (p.Ala80Thr)	Possibly damaging with a score of 0.85	Disease causing with a p value of 1	25.3	Homozygous	Known	Homozygous individual not known (1/105,640 alleles; only heterozygous individual known)	1,4,11,24
8	9	INAD	ND							
9	10	INAD	c.671T>C (p.Leu224Pro)	Probably damaging with a score of 1	Disease causing with a p value of 0.99	31	Homozygous	Novel	Not present	Present study
10	11	INAD	ND							
11	12	ANAD	ND							
12	13	INAD	c.2030G>T (p.Arg677Leu)	Possibly damaging with a score of 0.83	Disease causing with a p value of 0.86	22.4	Heterozygous	Known	Homozygous individual not known (13/109,150 alleles; only heterozygous individual known)	Present study
13	14	ANAD	ND							
14	15	INAD	c.847G>A (p.Asp283Asn)	Probably damaging with a score of 1	Disease causing with a p value of 0.99	9.93	Homozygous	Novel	Not present	Present study
15	16	INAD	ND							
16	18	ANAD	ND							
17	20	INAD	c.1471C>T (p.Leu491Phe)	Probably damaging with a score of 1	Disease causing with a p value of 0.99	26.8	Homozygous	Novel	Not present	Present study
18	20	INAD	c.1547C>T (p.Ala516Val)	Possibly damaging with a score of 0.77	Possibly damaging with a score of 0.77	26.8	Homozygous	Known	Homozygous individual not known (1/20,836 alleles; only heterozygous individual known)	Present study
19	21	ANAD	ND							
20	22	DPC	ND							
21	23	INAD	c.1613G>A (p.Arg538His)	Possibly damaging with a score of 0.89	Disease causing with a p value of 0.99	27.1	Homozygous	Known	Homozygous individual not known (1/120,738 alleles; only heterozygous individual known)	Present study
22	24	ANAD	c.1912G>A (p.Gly638Arg)	Probably damaging with a score of 1	Disease causing with a p value of 0.99	22.9	Compound heterozygous	Known	Not present	1,4
23	24	ANAD	c.1946G>A (p.Arg649His)	Probably damaging with a score of 1	Disease causing with a p value of 0.99	23.1		Novel	Not present	Present study
24	25	ANAD	ND							

ND, mutation not detected in *PLA2G6*.

Of 13 mutations, only p.Leu224Pro was recurrent and observed in a heterozygous state in family 2 and in a homozygous state in family 10 (Table 2). Of the remaining 12 mutations, one mutation p.Arg677Leu (family 13) was identified in a heterozygous state, nine mutations (viz., p.Arg70*, p.Ala80Thr, p.Asp283Asn, p.Arg329Cys, p.Gly347Arg, p.Leu491Phe, p.Ala516Val, p.Arg538His, and p.Arg741Trp) were observed in a homozygous state, and the remaining two mutations, p.Arg649His and p.Gly638Arg, were observed in a compound heterozygous state in family 24 (Table 2). Interestingly, the affected individual II-2 from family 20 was homozygous for two novel mutations, p.Leu491Phe and p.Ala516Val (Fig C in S1 File), and thus harboured four mutant alleles.

A spectrum of neurodegenerative disorders with mutations in *PLA2G6* is called ‘‘PLA2G6-associated neurodegeneration” (PLAN), which is a heterogeneous group of neurodegenerative condition representing INAD, ANAD and DPC. To date, 121 mutations (Table C in S1 File) in *PLA2G6* have been reported in patients with INAD, ANAD and DPC worldwide [4–30]. We found *PLA2G6* mutations in INAD and ANAD patients only (Table C in S1 File). With novel mutations identified during the present study, the total number of mutations in this gene reaches 129 (Table C in S1 File). These mutations are scattered throughout the exons of *PLA2G6*, suggesting that its entire coding region needs to be sequenced to find mutations in the patients (Table C in S1 File).

As stated above, we were not successful in identifying mutations in 10/22 families, suggesting that the mutations may reside in the promoter or deep intronic regions of this gene, which we have not sequenced, or these families could have mutations in a yet to be identified gene. Or these families may have differential diagnosis of similar clinical presentations, which include other causes of neurodegeneration with brain iron accumulation such as atypical Pantothenate kinase associated neurodegeneration (PKAN), Kufor-Rakeb syndrome and Mitochondrial membrane protein-associated neurodegeneration [33]. Similar to our observation, Gregory et al. [1] have screened 73 families for mutations in this gene and could not identify mutations in 27 families. Further, Zhang et al. [24] could not identify mutations in 2/25 families from China. Moreover, Morgan et al. [4] have suggested that INAD is genetically heterogeneous, as their linkage data supported the existence of at least one additional INAD locus. Of 12/22 families with *PLA2G6* mutations, two families (viz., family 2 and 13) were heterozygous for a single mutant allele, without a second mutation. Other studies have also identified heterozygous mutations in this gene [7–8, 11, 24]. Our inability to find the second mutation in these families could be due to the presence of large deletions, duplications and insertions, which are not amenable to Sanger sequencing.

**Fig 4:**
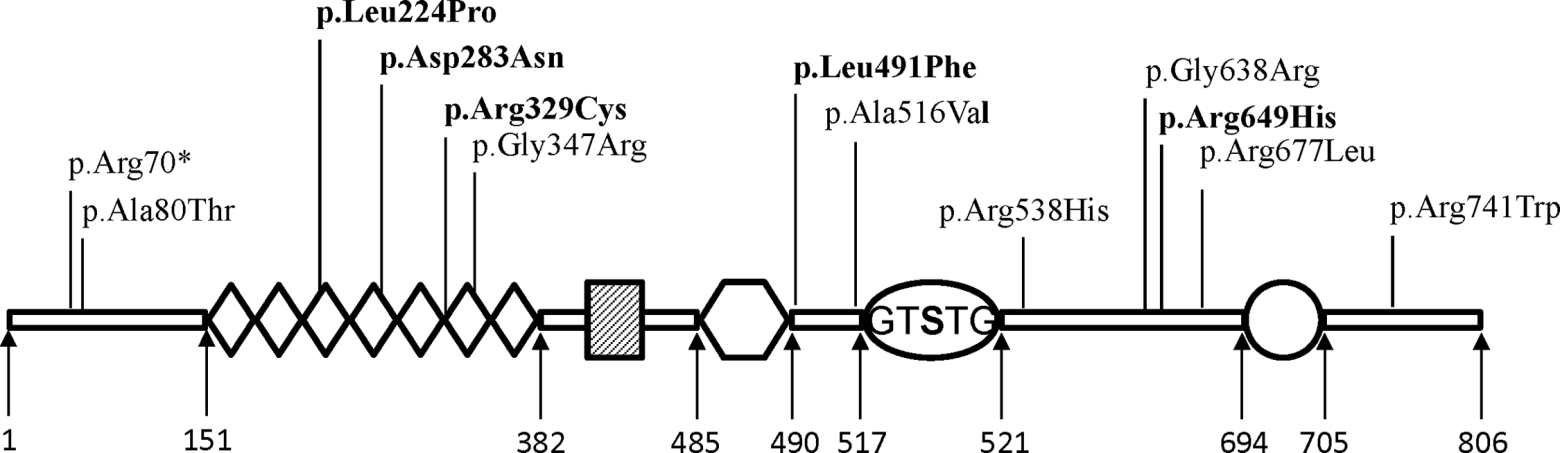
Schematic representation of PLA2G6 and location of mutations identified in present study. PLA2G6 consists of seven ankyrin repeats (diamond), a proline-rich motif (gridded box), a nucleotide binding motif (hexagon), a lipase motif (oval) and a binding site for calmodulin (circle). Numbers shown below are the amino acid positions. Five novel mutations are shown in bold.

The PLA2G6 (iPLA2-VIA) protein harbours the following domains from the N-terminus to C-terminus: ankyrin repeats, proline-rich motif, glycine-rich nucleotide binding motif, lipase motif and calmodulin binding site (Fig 4). The glycine-rich nucleotide binding motif, a lipase motif (GTSTG) and a binding site for calmodulin, all contribute to the regulation of the enzymatic activity. The seven ankyrin-like repeats are involved in oligomerization of the enzyme, which is a crucial step for full enzymatic activity [3]. Deficiency of PLA2G6 activity may disrupt membrane homeostasis by altering the phospholipid composition of cellular and sub-cellular membranes leading to structural damage [34–35]. It also leads to changes in membrane permeability, fluidity or ion homeostasis and may cause accumulation of membranes, organelles, and protein in distal axons that represent spheroids [34–35]. A direct connection between defects in PLA2G6 and development of the neurodegenerative process is not understood yet. However, on the basis of the relevant role of PLA2G6 in maintaining membrane phospholipid homeostasis, it could be speculated that the perturbation of membrane lipid homeostasis may lead to structural abnormalities and may underlie axonal degeneration, which is the essential pathological hallmark of INAD [8, 11].

A mouse model with an amino acid substitution in the ankyrin repeat domain of PLA2G6 has provided the evidence of motor impairment at the age of 2 months, which progressively worsened [36]. The mice died between 18 and 24 weeks of age, and the pathology was characterized by the presence of numerous spheroids, very similar to those observed in INAD [36]. We speculate that the three novel missense mutations (viz., p.Leu224Pro, p.Asp283Asn and p.Arg329Cys) located in the ankyrin repeat domains may interfere with the oligomerization of the enzyme. Since all the novel missense mutations reported in this study occurred at amino acid positions that are highly conserved, we speculate that they could be important for the normal functioning of the protein. However, further functional studies are necessary to illustrate the exact pathogenesis due to these mutations.

Even though no clear genotype/phenotype correlation exists, the early onset and more aggressive forms of INAD are associated with truncating *PLA2G6* mutations, while the ‘atypical’ presentation is more common in individuals with missense mutations [24]. Interestingly, a majority (85/129) of the mutations reported here are missense (Table C in S1 File). Engel et al. [37] have demonstrated that the catalytic function of PLA2G6 was impaired by mutations associated with INAD, but not with DPC. However, further functional studies are required to elucidate the exact pathogenesis caused by mutations in this gene.

In conclusion, we have identified a total of 13 different mutations in *PLA2G6*, including five novel ones, in 12/22 families. The absence of mutations in the remaining 10/22 families suggests that the mutations could be in deep intronic or promoter regions of this gene or these families have large deletions, which are not amenable to Sanger sequencing. It is also possible that INAD in these families is due to mutations in a yet to be identified gene. Further, with the five novel mutations reported here, the total number of mutations described to date in *PLA2G6* reaches 129 (Table C in S1 File). The present finding will be useful for genetic diagnosis, carrier detection and genetic counselling to families included in this study and other families with similar disease condition.

## Supporting Information

S1 File Supporting multiple Figures and Tables (pdf). (PDF)
