# Supplementary material for: Genetic Analysis of PLA2G6 in 22 Indian Families with Infantile Neuroaxonal Dystrophy, Atypical Late-Onset Neuroaxonal Dystrophy and Dystonia Parkinsonism Complex
Source: PLoS One. 2016 May 19;11(5):e0155605. doi: 10.1371/journal.pone.0155605 (PMC4873246; doi:10.1371/journal.pone.0155605)
Supplement: S1 File — (PDF) [file pone.0155605.s001.pdf]

File S1: Supporting multiple Figures and Tables

Genetic analysis of *PLA2G6* in 22 Indian families with infantile neuroaxonal dystrophy, atypical late-onset neuroaxonal dystrophy and dystonia parkinsonism complex

Saketh Kapoor[1]¶, Mohd Hussain Shah[1]¶, Nivedita Singh[1]¶, Mohammad Iqbal Rather[1], Vishwanath Bhat[1], Sindhura Gopinath[2], Parayil Sankaran Bindu[3*], Arun B. Taly[3], Sanjib Sinha[3], Madhu Nagappa[3], Rose Dawn Bharath[4], Anita Mahadevan[5], Gayathri Narayanappa[5], Yasha T. Chickabasaviah[5], Arun Kumar[1*]

[1]Department of Molecular Reproduction, Development and Genetics, Indian Institute of Science, Bangalore 560012, India.

[2]Department of Biotechnology, R.V. College of Engineering, Bangalore 560059, India.

[3]Department of Neurology, National Institute of Mental Health and Neuro Sciences, Bangalore 560029, India.

[4]Department of Neuroimaging and Interventional Radiology, National Institute of Mental Health and Neuro Sciences, Bangalore 560029, India.

[5]Department of Neuropathology, National Institute of Mental Health and Neuro Sciences, Bangalore 560029, India.

* Corresponding authors

E-mails: karun@mrdg.iisc.ernet.in (AK) and drpsbindu@yahoo.co.in (PSB)

¶ These authors contributed equally to this work.

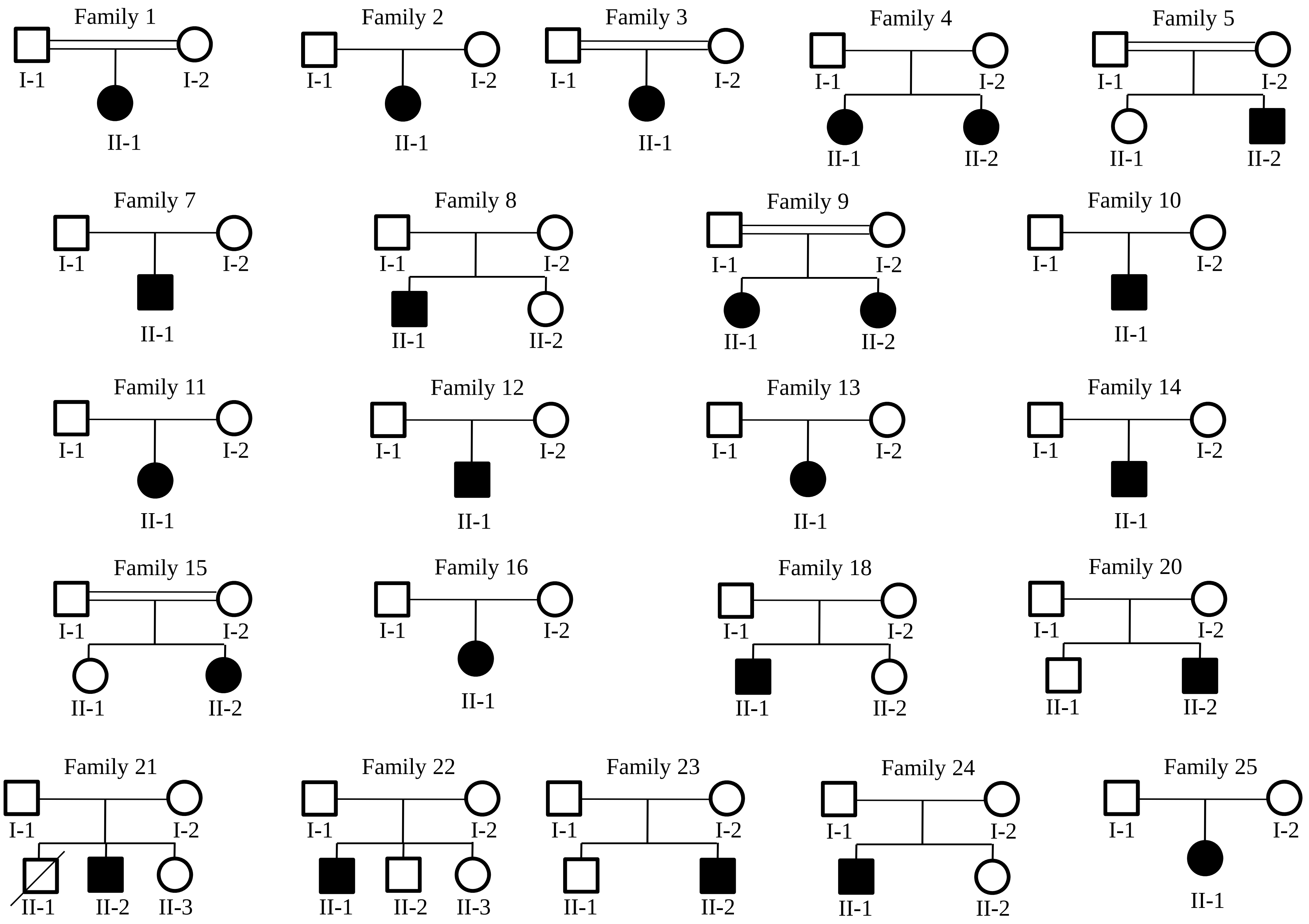

Fig A. Pedigrees of PLAN families.

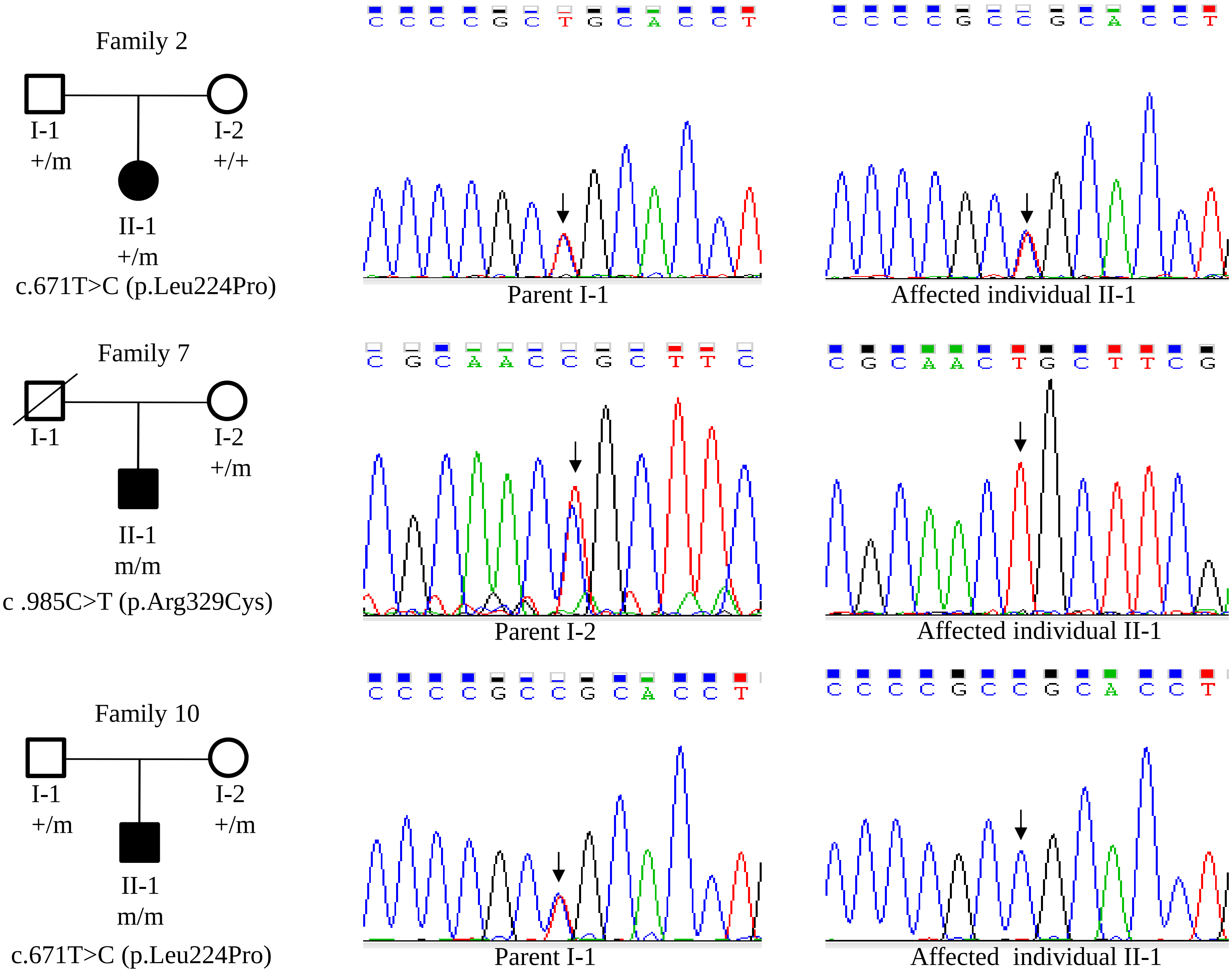

Fig B. DNA sequence analysis of individuals. (Upper panel) Sequencing chromatograms from the parent I-1 and the affected individual II-1 from family 2. Arrows mark the T>C change in a heterozygous state in the parent I-1 and the affected individual II-1. (Middle panel) Sequencing chromatograms from the parent I-2 and the affected individual II-1 from family 7. Arrows mark the C>T change in a heterozygous state in the parent I-2 and in a homozygous state in the affected individual II-1. (Lower panel) Sequencing chromatograms from the parent I-1 and the affected individual II-1 from family 10. Arrows mark the T>C change in a heterozygous state in the parent I-1 and in a homozygous state in the affected individual II-1. + and m denote the wild type and mutant alleles, respectively.

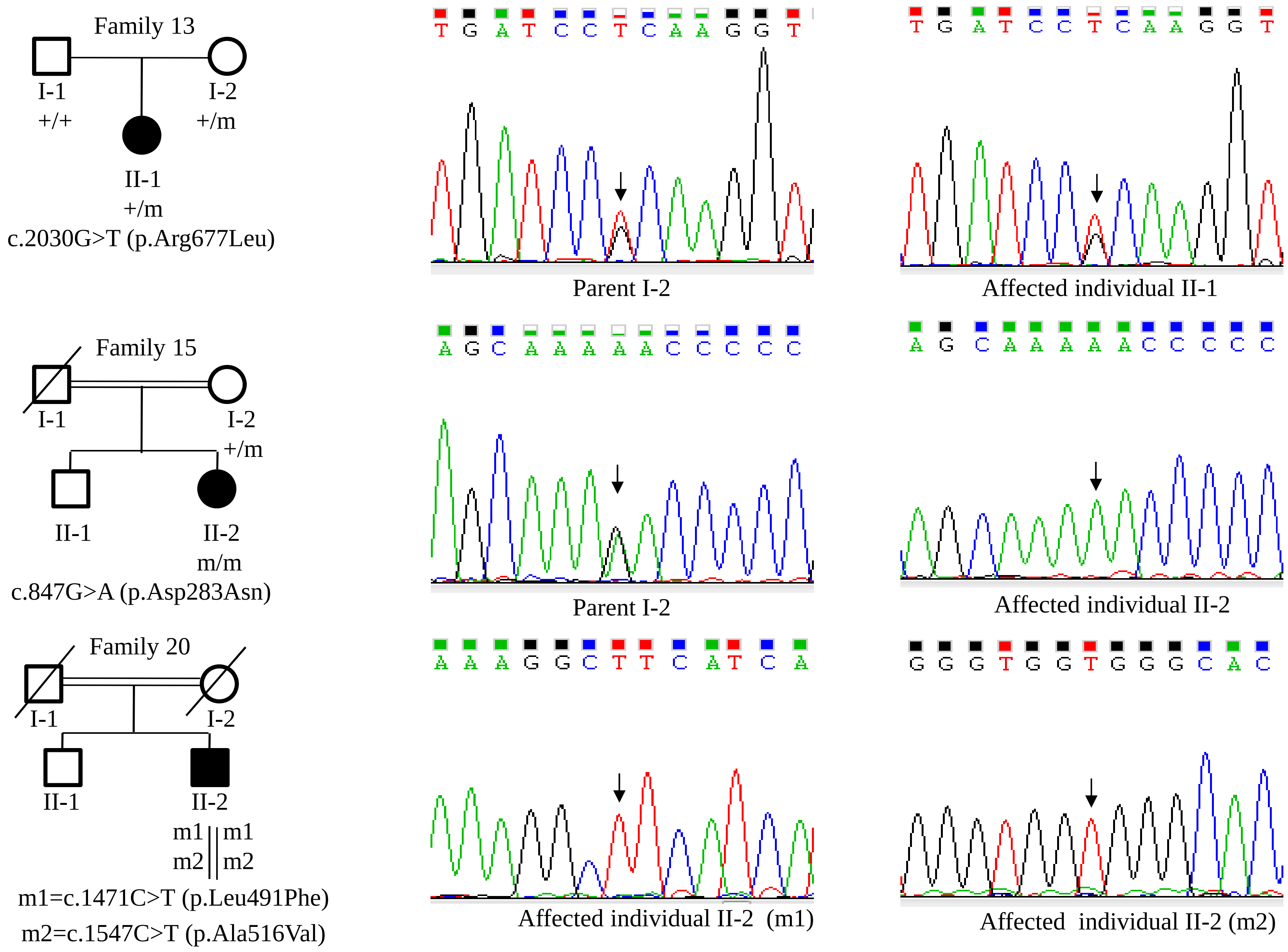

Fig C. DNA sequence analysis of individuals. (Upper panel) Sequencing chromatograms from the parent I-2 and the affected individual II-1 from family 13. Arrows mark the G>T change in a heterozygous state in the parent I-2 and the affected individual II-1. (Middle panel) Sequencing chromatograms from the parent I-2 and the affected individual II-2 from family 15. Arrows mark the G>A change in a heterozygous state in the parent I-2 and in a homozygous state in the affected individual II-2. (Lower panel) Sequencing chromatograms from the affected individual II-2 from family 20. Arrows mark the C>T change in a homozygous state in the affected individual II-2. Note, this individual is homozygous for two different mutations. + and m denote the wild type and mutant alleles, respectively. m1 and m2 denote different mutant alleles.

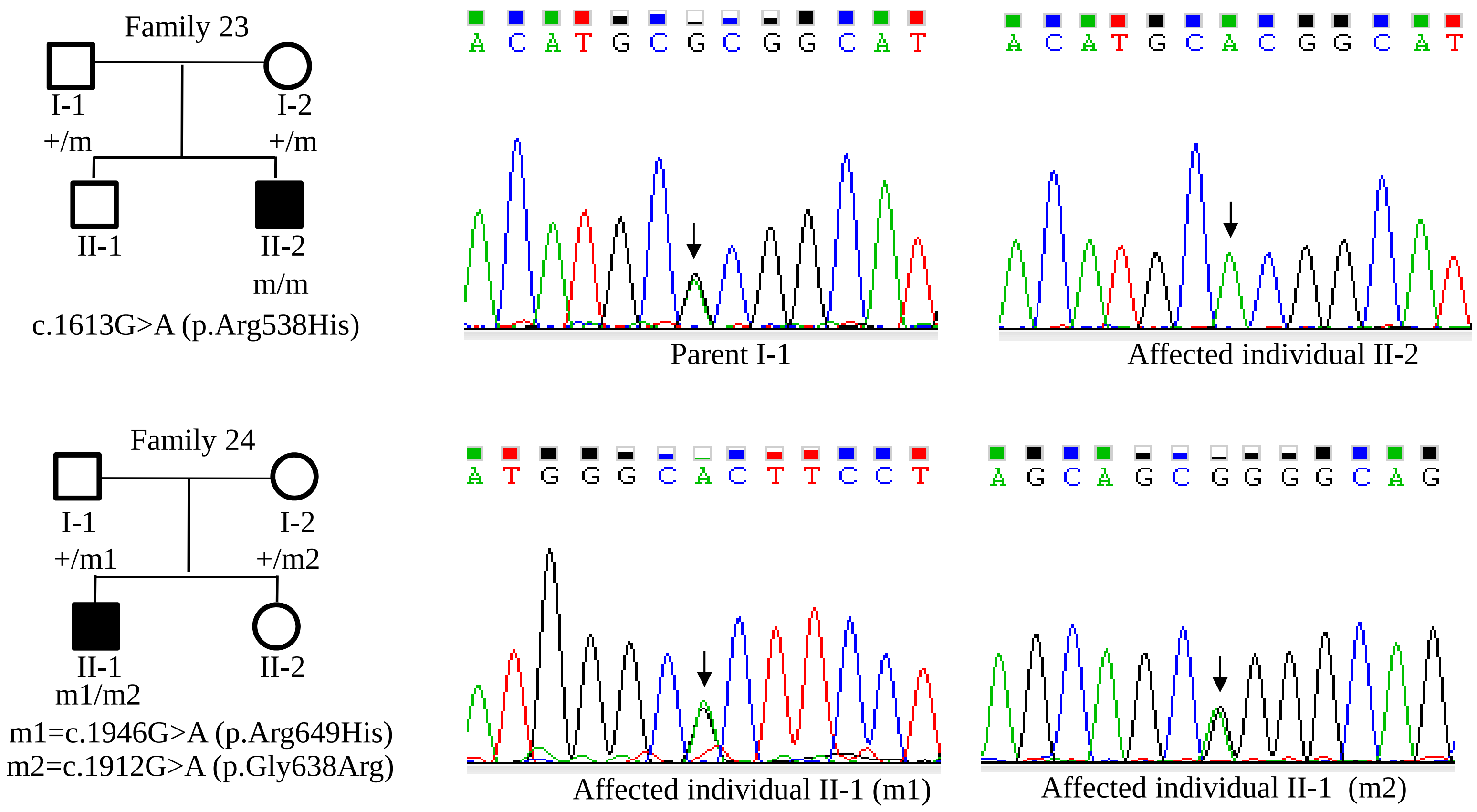

Fig D. DNA sequence analysis of individuals. (Upper panel) Sequencing chromatograms from the the parent I-1 and the affected individual II-2 from family 23. Arrows mark the G>A change in a heterozygous state in the parent I-1 and in a homozygous state the affected individual II-2. + and m denote the wild type and the mutant alleles, respectively. (Lower panel) Sequencing chromatograms from the affected individual II-1 from family 24. Arrows mark the G>A change in a heterozygous state in the affected individual II-1. m1 and m2 denote different mutation alleles.

Table A. Details of PCR primers used in the mutation analysis of the *PLA2G6* gene.

Sl. #	Exon	Primer Sequence (5' to 3')	T_m (°C)	Amplicon size (bp)
1	2	F:AGTGATCTGGGTGTCTGTGCAGGA<br>R:CTCTCCAGACCCCTCAGACAGAGA	64	465
2	3	F:ACCTTCTGATTCCAGCAGGGATGTG<br>R:AGTGCTGGGACTGCGTTAGTGAGC	64	445
3	4	F:CCATCTCTTGTACGAGTGAGCAAAC<br>R:GTTGGAGGCCGTCCCCAGGTTTA	64	647
4	5	F:GCCCGGCCTCTTTACGTTCTTATC<br>R:GAGGCTTGTGAACCCGGGGCCT	64	394
5	6	F:AGCAGTAGATGAAGCTCCAAGTCCTG<br>R:ACTTTACAATTTGTGCCGAGTTTTGTCAC	64	485
6	7	F:TGCTAGCGCTTTCCAACATCCCA<br>R:AGCAGCTGACGATAGGAGGGAGA	64	418
7	8	F:TCCCCTGTGGGTGCTTTTAAGCCTG<br>R:GTCAGCACAGGATGCTGGCACCTG	64	441
8	9	F:GCTGGGACAGGGGACGGAGTGTG<br>R:ACTTCCGTCCTAGGGATCCTGTTGC	64	361
9	10	F:TGGCCGTGGGCGAGGCACAGC<br>R:GCATTAATGAACGAGCGACACAGGC	72	369
10	11	F:GGACAGAAGTTCCTCGGGTCCGTC<br>R:TTAAGGCAGTCGATAGGAAACGCTTC	69	584
11	12	F:CTGCGTTCAGGCGCTCTGCAGGC<br>R:CCTCAAGCGTGGGGCGCTCTTCC	64	352
12	13	F:GGAGAAGCCCACCTATCCCGAACA<br>R:TGATGGCAAGTGCACGACTCCCAC	66	359
13	14	F:CCACCAGGACGAACTAGCCAGAGA<br>R:CTGTCCTGGGGGTCGGTCCCTAG	66	422
14	15	F:TGATGCCCGCACCAGCCGTTCTG<br>R:CAGCTGGCTAAAGGCGATGGACA	66	500
15	16	F:CTCAGCCTGACTCGAAAGAGCCTG<br>R:TGGGAGGGGAAGGTCGGTGAGTC	66	272
16	17	F:TGCCCTGGCGTGGTCGGACTCAC<br>R:GTGCCCGGGTACCCTTAATGTTTGA	66	616

Abbreviations: F, forward primer; R, reverse primer; bp, base pairs; and Tm, Annealing temperature.

Table B. Details of primers used in allele-specific PCR.

S.No.	Mutation	Primer Sequence (5’ to 3’)	T_m (°C)	Amplicon size (bp)
1	c.671T>C (p.Leu224Pro)	F:ATAACCAAGGGCTGACCCCGCC	64	246
		R:GAGGCTTGTGAACCCGGGGCCT		
2	c.847G>A (p.Asp283Gln)	F:GGACAGCAGCCAGATCCACAGCAAAA	70	317
		R:ACTTTACAATTTGTGCCGAGTTTTGTCAC		
3	c.985C>T (p.Arg329Cys)	F:CACGTGGCGGTGATGCGCAACT	64	178
		R:AGCAGCTGACGATAGGAGGGAGA		
4	c.1471C>T (p.Leu491Phe)	F:TGGAGGAGGAGTGAAAGGCT	60	216
		R:CTTATAGCCCTCCTCTACTCC		
5	c.1613G>A (p.Arg538His)	F:TAAGTCCATGGCCTACATGCA	66	116
		R:GGGCTTACTTGGGTTTCCTGA		
6	c.1946G>A (p.Arg649His)	F:TACTTCCGACCCAATGGGCA	66	157
		R:CTGGGGGTCGGTCCCTAG		

Abbreviations: F, forward primer; R, reverse primer; Tm, annealing temperature; and, bp, base pairs.

Table C. Known mutations in *PLA2G6*.

Sl. #	Mutation	Exon/ intron (IVS)	Nature of mutation	State of zygosity	Effect on protein	Disease	Number and ethnic origin of family	Reference
1	c.1A>G (p.Met1Val)	2	Missense	Heterozygous or compound heterozygous with c.109C>T (p.Arg37*) or c.1111G>A (p.Val371Met)	Defect in function of protein if leads to abnormal translation	INAD	6 Chinese	11, 24
2	c.4C>A (p.Gln2Lys)	2	Missense	Compound heterozygous with deletion of exon 3	Inactivates protein	ANAD	1 French	16
3	c.27_28insA (p.Thr10Asnfs*9)	2	Insertion	Compound heterozygous with c.668C>A (p.Pro223Gln)	Premature protein truncation	INAD	1 Chinese	24
4	c.109C>T (p.Arg37*)	2	Nonsense	Homozygous or compound heterozygous with c.1A>G (p.Met1Val),c.609_894del286 (p.Leu204_Glu298del95), c.692G>T (p.Gly231Val), IVS8 -3C>Aorc.1501G>C (p.Glu501Gln)	Premature protein truncation	INAD & DPC	4 British, 3 American, 3 Chinese, 1 Pakistani & 1 Italian	1, 3, 9, 11, 14, 21, 24
5	c.116G>T (p.Arg39Gln)	2	Missense	Compound heterozygous with c.1771C>T (p.Arg591Trp)	Inactivates protein	INAD	2 Chinese	11, 24
6	c.171C>A (p.Cys57*)	2	Nonsense	Homozygous	Premature protein	INAD	1 Chinese	24

					truncation			
7	c.208C>T (p.Arg70*)	2	Nonsense	Homozygous or heterozygous	Truncated protein or mRNA degradation by nonsense-mediated mRNA decay	INAD	1 Chinese & 1 Indian	11, Present study
8	Exon 3 deletion	3	Deletion	Compound heterozygous with c.4C>A (p.Gln2Lys)	Inactivates protein	ANAD	1 French	16
9	c.216C>A (p.Phe72Leu)	3	Missense	Compound heterozygous with c.1904G>A (p.Arg635Gln)	Inactivates protein	DPC	1 Japanese	15
10	c.238G>A (p.Ala80Thr)	3	Missense	Homozygous or compound heterozygous with c.2370_2371delTG (p.Tyr790*) orc.1970C>T (p.Ala657Val)	Inactivates protein	INAD & ANAD	1 British, 2 Chinese, 1 American & 1 Indian	1, 3, 11, 24, Present study
11	c.317delT (p.Val106fs*110)	3	Deletion	Homozygous	Premature protein truncation	INAD	1 American	1
12	c.319delC (p.Leu107fs*110)	3	Deletion	Heterozygous	Premature protein truncation	ANAD & DPC	1 British, 1 American& 1 American Caucasian	1, 3, 21
13	c.369_377dup (p.His124_Ala126 dup)	3	Duplication	Compound heterozygous with c.1903C>T (p.Arg635*)	Inactivates protein	INAD	1 Tunisian	30
14	c.372delC (p.His124Leufs*3 24)	3	Deletion	Compound heterozygous with c.2261G>T (p.Gly754Val)	Premature protein truncation	ANAD	1 Chinese	24

15	c.404T>C (p.Phe135Ser)	3	Missense	Compound heterozygous with c.1472T>G (p.Leu491Arg)	Inactivates protein	INAD	1 British & 1 American	1, 3
16	c.439G>A (p.Ala147Thr)	4	Missense	Compound heterozygous with c.2132C>T (p.Pro711Leu)	Inactivates protein	INAD	1 British & 1 American	1, 3
17	c.469C>T (p.His157Tyr)	4	Missense	Homozygous	May interfere with the oligomerization	INAD	1 British &1 American	1, 3
18	c.470A>G (p.His157Arg)	4	Missense	Compound heterozygous with c.1021G>A (p.Ala341Thr) or c.1903C>T (p.Arg635*)	May interfere with the oligomerization	INAD	2 British &2 American	1, 3
19	Duplication of exons 4-7	4-7	Duplication	Compound heterozygous with c.1674delG (p.Leu560Trpfs*5)	May interfere with the oligomerization	INAD	2 Caucasian	12, 28
20	IVS5 -1G>T	5	Splice site	Compound heterozygous with c.2370delTG (p.Tyr790*)	Premature protein truncation	INAD & DPC	1 American	21
21	c.609_894del286 (p.Leu204_Glu298del95)	5-6	Deletion	Compound heterozygous with c.109C>T (p.Arg37*)	May interfere with the oligomerization	INAD	1 Italian	14
22	c.665C>T (p.Thr222Ile)	5	Missense	Heterozygous	May interfere with the oligomerization	INAD	1 Caucasian of western European descent &1 American	1, 8
23	c.668C>A (p.Pro223Gln)	5	Missense	Homozygous or compound heterozygous with c.27_28insA	May interfere with the oligomerization	INAD & ANAD	2 Chinese & 1 American	1, 24

				(p.Thr10Asnfs*9)				
24	c.671T>C (p.Leu224Pro)	5	Missense	Homozygous or Heterozygous	May interfere with the oligomerization	INAD	2 Indian	Present study
25	c.673C>T (p.His225Tyr)	5	Missense	Homozygous	May interfere with the oligomerization	ANAD	1 Saudi Arabian	25
26	c.691G>C (p.Gly231Arg)	5	Missense	Compound heterozygous with c.2370T>G (p.Tyr790*)	May interfere with the oligomerization	ANAD	1 Caucasian	28
27	c.692G>T (p.Gly231Val)	5	Missense	Compound heterozygous with c.109C>T (p.Arg37*)	May interfere with the oligomerization	INAD	1 Chinese	24
28	c.719T>C (p.Leu240Pro)	5	Missense	Compound heterozygous with c.1978C>A (p.Pro660Thr)	May interfere with the oligomerization	INAD	1 Arabic	9
29	c.751_759del9bp (p.Pro251_Gly253del)	5	Deletion	Compound heterozygous with c.1463T>G (p.Val488Gly)	May interfere with the oligomerization	INAD	1 British & 1 American	1, 3
30	c.755delA (p.Asn252fs*260)	5	Deletion	Compound heterozygous with c.1061T>C (p.Leu354Pro)	Premature protein truncation	INAD	1 British& 1 American	1, 3
31	Exon5 or 6 deletion	5-6	Deletion	Homozygous	May interfere with the oligomerization	INAD	2 Irish	12
32	c.847G>A (p.Asp283Asn)	6	Missense	Homozygous	May interfere with oligomerization	INAD	1 Indian	Present study
33	c.848A>G (p.Asp283Gly)	6	Missense	Homozygous	May interfere with the oligomerization	INAD	1 British & 1 American	1, 3
34	c.858C>G (p.Thr286*)	6	Nonsense	Heterozygous	Premature protein	INAD	1 Chinese	24

					truncation			
35	IVS7 -1G>A	IVS7	Splice site	Compound heterozygous with IVS8 +1G>A	Premature protein truncation	INAD	1 Italian	14
36	c.901C>T (p.Arg301Cys)	7	Missense	Heterozygous	May interfere with the oligomerization	DPC	1 Japanese	19
37	c.905T>G (p.Met302Arg)	7	Missense	Homozygous	May interfere with the oligomerization	INAD	1 Pakistani	9
38	c.929T>A (p.Val310Glu)	7	Missense	Homozygous or compound heterozygous with IVS10 + 1G>A	May interfere with the oligomerization	INAD	1 British, 1 American & 1 Chinese	1, 3, 11
39	c.985C>T (p.Arg329Cys)	7	Missense	Homozygous	May interfere with oligomerization	INAD	1 Indian	Present study
40	c.991G>T (p.Asp331Tyr)	7	Missense	Homozygous or compound heterozygous with c.1077G>A (p.Met358Ilefs*)	Reduction in enzyme activity	DPC	3 Taiwanese & 1 Japanese	18, 20
41	c.991G>A (p.Asp331Asn)	7	Missense	Heterozygous	May interfere with the oligomerization	DPC	1 Japanese	19
42	c.1018G>A (p.Gly340Arg)	7	Missense	Homozygous	May interfere with the oligomerization	INAD	1 British & 1 American	1, 3
43	c.1021G>A (p.Ala341Thr)	7	Missense	Compound heterozygous with c.470A>G (p.His157Arg)	May interfere with the oligomerization	INAD	1 British & 1 American	1, 3
44	c.1039G>A (p.Gly347Arg)	7	Missense	Homozygous	May interfere with the oligomerization	INAD	2 American, 1 British & 1 Indian	1, 3, Present study
45	c.1058C>T (p.Pro353Leu)	7	Missense	Compound heterozygous with c.1549G>T	May interfere with the oligomerization	INAD	1 British & 1 American	1, 3

				(p.Gly517Cys)				
46	c.1061T>C (p.Leu354Pro)	7	Missense	Compound heterozygous with c.755delA (p.Asn252fs*260), c.1933C>T (p.Arg654*) or c.2215G>C (p.Asp739His)	May interfere with the oligomerization	INAD, ANAD & DPC	2 British & 3 American	1, 3, 21
47	c.1077G>A (p.Met358Ilefs*6)	7	Frameshift	Heterozygous or compound heterozygous with c.991G>T (p.Asp331Tyr) or IVS10 + 1G>A	Premature protein truncation	INAD & DPC	3 Chinese & 2 Taiwanese	20, 24
48	c.1103C>A (p.Ala368Asp)	7	Missense	Compound heterozygous with c.1417G>T (p.Glu473*)	May interfere with the oligomerization	INAD	1 British	9
49	IVS8 -3C>A	IVS8	Splice site	Compound heterozygous with c.109C>T (p.Arg37*)	Premature protein truncation	INAD & DPC	1 British	21
50	c.1111G>A (p.Val371Met)	8	Missense	Compound heterozygous with c.1A>G (p.Met1Val)	May interfere with the oligomerization	INAD	2 Chinese	11, 24
51	c.1117G>A (p.Gly373Arg)	8	Missense	Compound heterozygous with c.1633A>G (p.Lys545Glu) or c.2065A>T (p.Ile689Phe)	May interfere with oligomerization	INAD	3 Chinese	11, 24
52	c.1125delA (p.Glu376Trpfs*14)	8	Deletion	Homozygous	Predicted to lead to the nonsense mediated	INAD	1 Saudi Arabian	25

					mRNA decay			
53	IVS8 +1G>A	IVS8	Splice site	Compound heterozygous with IVS7 -1G>A	Defect in function of protein due to aberrant splicing	INAD	1 Italian	14
54	IVS9 -2A>G	IVS9	Splice site	Compound heterozygous with c.1612C>T (p.Arg538Cys)	Defect in function of protein due to aberrant splicing	ANAD	1 Japanese	26
55	c.1351delC (p.Leu451fs*470)	10	Deletion	Homozygous	Premature protein truncation	INAD	2 British & 1 American	1, 3
56	c.1351delC (p.Leu451Tyrfs*20)	10	Deletion	Homozygous	Premature protein truncation	INAD	1 Portuguese	27
57	c.1354C>T (p.Gln452*)	10	Nonsense	Compound heterozygous with c.1904G>A (p.Arg635Gln)	Premature protein truncation	DPC	1 Japanese	15
58	c.1417G>T (p.Glu473*)	10	Nonsense	Compound heterozygous with c.1103C>A (p.Ala368Asp)	Premature protein truncation	INAD	1 British	9
59	IVS10 +1G>A	IVS10	Splice site	Homozygous, heterozygous or compound heterozygous with c.929T>A (p.Val310Glu) or c.1077G>A (p.Met358Ilefs*6)	Defect in function of protein due to aberrant splicing	INAD	5 Chinese	11, 24
60	c.1442T>A (p.Leu481Gln)	11	Missense	Homozygous or compound heterozygous with c.2070_2072delTGT (p.Val691del) or	Inactivates protein	INAD	2 British, 2 American, 1 Caucasian of western European	1, 3, 8, 29,30

				c.2370T>G (p.Tyr790*)			descent, 1 Tunisian & 1 Moroccan	
61	c.1463T>G (p.Val488Gly)	11	Missense	Compound heterozygous with c.751_759del9bp (p.Pro251_Gly253del)	Inactivates protein	INAD	1 British & 1 American	1, 3
62	c.1471C>T (p.Leu491Phe)	11	Missense	Homozygous with c.1547C>T (p.Ala516Val)	Inactivates protein	INAD	1 Indian	Present study
63	c.1472T>G (p.Leu491Arg)	11	Missense	Compound heterozygous with c.404T>C (p.Phe135Ser)	Inactivates protein	INAD	1 British & 1 American	1, 3
64	c.1496C>T (p.Ala499Val)	11	Missense	Compound heterozygous with c.1903C>T (p.Arg635*)	Inactivates protein	INAD	1 Chinese	24
65	c.1501G>C (p.Glu501Gln)	11	Missense	Compound heterozygous with c.109C>T (p.Arg37*)	Inactivates protein	INAD	1 British & 1 American	1, 3
66	c.1506G>C (p.Lys502Asn)	11	Missense	Compound heterozygous with c.1612C>T (p.Arg538Cys) or c.1903C>T (p.Arg635*)	Inactivates protein	INAD	2 American & 1 British	1, 3
67	c.1524dupC (p.Leu509Glnfs*5)	11	Duplication	Compound heterozygous with c.1798C>T (p.Arg600Trp)	Premature protein truncation	INAD	1 Caucasian	30
68	c.1538_1541delA CTG (p.Asp513fs*543)	11	Deletion	Compound heterozygous with c.2370_2371delTG (p.Tyr790*)	Premature protein truncation	INAD	1 British & 1 American	1, 3
69	c.1547C>T	11	Missense	Homozygous with	Inactivates	INAD	1 Indian	Present study

	(p.Ala516Val)			c.1471C>T (p.Leu491Phe)	protein			
70	c.1549G>T (p.Gly517Cys)	11	Missense	Compound heterozygous with c.1058C>T (p.Pro353Leu)	Inactivates protein	INAD	1 British & 1 American	1, 3
71	c.1610T>A (p.Met537Lys)	12	Missense	Homozygous	Inactivates protein	INAD	1 Chinese	24
72	c.1612C>T (p.Arg538Cys)	12	Missense	Compound heterozygous with IVS9 -2A>G or c.1506G>C (p.Lys502Asn)	Inactivates protein	ANAD & INAD	1 British, 1 American & 1 Japanese	1, 3, 26
73	c.1613G>A (p.Arg538His)	12	Missense	Homozygous	Inactivates protein	INAD	1 Indian	Present study
74	c.1633A>G (p.Lys545Glu)	12	Missense	Heterozygous or compound heterozygous with c.1117G>A (p.Gly373Arg)	Inactivates protein	INAD	3 Chinese	11, 24
75	c.1634A>C (p.Lys545Thr)	12	Missense	Homozygous	Inactivates protein	ANAD	5 Pakistani	3, 9
76	c.1776A>G (p.Glu547Gly)	12	Missense	Homozygous	Inactivates protein	ANAD	1 Tunisian	30
77	c.1674delG (p.Leu560Trpfs*5)	12	Deletion	Compound heterozygous with duplication of exons 4-7	Premature protein truncation	INAD	2 Caucasian	12, 28
78	c.1699G>A (p.Glu567Lys)	12	Missense	Compound heterozygous with c.1754C>T (p.Thr585Ile)	Inactivates protein	INAD	1 British & 1 American	1, 3
79	c.1703T>G (p.Phe568Val)	12	Missense	Homozygous	Inactivates protein	INAD	1 Tunisian	30
80	c.1715 C>T (p.Thr572Ile)	12	Missense	Homozygous	Inactivates protein	ANAD & DPC	1 Greek	21
81	c.1744G>T	13	Missense	Compound	Inactivates	INAD &	1 British & 1	1, 3

	(p.Val582Leu)			heterozygous with c.2233C>T (p.Arg745Trp)	protein	ANAD	American	
82	c.1754C>T (p.Thr585Ile)	13	Missense	Compound heterozygous with c.1699G>A (p.Glu567Lys)	Inactivates protein	INAD	1 British & 1 American	1, 3
83	c.1756G>A (p.Gly586Arg)	13	Missense	Homozygous	Inactivates protein	INAD	1 Caucasian	28
84	c.1770C>T (p.Asp590Glu)	13	Missense	Compound heterozygous with c.2221C>T (p.Arg741Trp)	Inactivates protein	INAD	1 British & 1 American	1, 3
85	c.1771C>T (p.Arg591Trp)	13	Missense	Compound heterozygous with c.116G>T (p.Arg39Gln) or c.1970C>T (p.Ala657Val)	Inactivates protein	INAD	3 Chinese	11, 24
86	c.1772G>A (p.Arg591Gln)	13	Missense	Homozygous	Inactivates protein	INAD	1 British, 1 American & 1 Saudi Arabian	1, 3, 25
87	c.1791delC (p.His597fs*69)	13	Deletion	Heterozygous	Reduced enzyme activity	DPC	1 Chinese	23
88	c.1798C>T (p.Arg600Trp)	13	Missense	Compound heterozygous with c.1524dupC (p.Leu509Glnfs*5)	Inactivates protein	INAD	1 Caucasian	28
89	c.1799G>A (p.Arg600Gln)	13	Missense	Homozygous	Inactivates protein	INAD & DPC	1 Hispanic	21
90	c.PLA2G6IVS13_ FLJ22582IVS2del (Fusion protein)	14	Deletion	Homozygous	Inactivates protein	INAD	2 British & 2 American	1, 3
91	c.1894C>T (p.Arg632Trp)	14	Missense	Homozygous or compound heterozygous with	Inactivates protein	ANAD & DPC	1 British & 1 Iranian	3, 10

				c.2070_2072delTGT (p.Val691del)				
92	c.1903C>T (p.Arg635*)	14	Nonsense	Homozygous or compound heterozygous with c.369_377dup (p.His124_Ala126dup), c.470A>G (p.His157Arg), c.1496C>T (p.Ala499Val) or c.1506G>C (p.Lys502Asn)	Premature protein truncation	INAD	4 American, 2 British, 2 Chinese & 1 Tunisian	1, 3, 24, 30
93	c.1904G>A (p.Arg635Gln)	14	Missense	Compound heterozygous with c.216C>A (p.Phe72Leu) or c.1354C>T (p.Gln452*)	Inactivates protein	DPC	2 Japanese	15
94	c.1911delC (p.Ser637Argfs*29)	14	Deletion	Homozygous	Predicted to lead to the nonsense mediated mRNA decay	INAD	1 Saudi Arabian	25
95	c.1912G>A (p.Gly638Arg)	14	Missense	Compound heterozygous with c.1946G>A (p.Arg649His) or c.2221C>T (p.Arg741Trp)	Inactivates protein	INAD	1 British, 1 American & 1 Indian	1, 3, Present study
96	c.1946G>A (p.Arg649His)	14	Missense	Compound heterozygous with c.1912G>A (p.Gly638Arg)	Inactivates protein	DPC	1 Indian	Present study
97	c.1959T>A (p.Gly653Gly)	14	Silent	Heterozygous	Could interfere with signals for RNA splicing or folding or	DPC	1 Chinese	23

					miRNA binding			
98	c.1957 G>A (p.Gly653Ser)	14	Missense	Compound heterozygous with c.2251G>T (p.Glu751*)	Inactivates protein	INAD	1 Chinese	24
99	c.1933C>T (p.Arg654*)	14	Nonsense	Homozygous or compound heterozygous with c.1061T>C (p.Leu354Pro)	Predicted to lead to the nonsense mediated mRNA decay	INAD & DPC	1 Saudi Arabian& 1 American caucasian	21, 25
100	c.1966C>G (p.Leu656Val)	14	Missense	Heterozygous	Reduced enzyme activity	DPC	1 Chinese	23
101	c.1970C>T (p.Ala657Val)	14	Missense	Compound heterozygous with c.238G>A (p.Ala80Thr) or c.1771C>T (p.Arg591Trp)	Inactivates protein	INAD	4 Chinese	11, 24
102	c.1976A>G (p.Asn659Ser)	14	Missense	Heterozygous	Inactivates protein	DPC	1 Taiwanese	20
103	c.1978C>A (p.Pro660Thr)	14	Missense	Compound heterozygous with c.719T>C (p.Leu240Pro)	Inactivates protein	INAD	1 Arabic	9
104	c.2030G>T (p.Arg677Leu)	15	Missense	Heterozygous	Inactivate protein	INAD	1 Indian	Present study
105	c.2036G>T (p.Gly679Val)	15	Missense	Heterozygous	Inactivate protein	DPC	1 Chinese	22
106	c.2060delT (p.Leu687Profs*16)	15	Deletion	Heterozygous	Premature protein truncation	INAD	1 Chinese	24
107	c.2065A>T (p.Ile689Phe)	15	Missense	Compound heterozygous with c.1117G>A (p.Gly373Arg)	Inactivates protein	ANAD	1 Chinese	24
108	c.2070_2072delT	15	Deletion	Homozygous or	Aberrant	INAD &	5 Tunisian, 2	1, 3, 4, 30

	GT (p.Val691del)			compound heterozygous with c.1442T>A (p.Leu481Gln) or c.1894C>T (p.Arg632Trp)	phospholipase activity	ANAD	British, 2 Israeli, 1 American & 1 Libyan	
109	c.2077C>G (p.Leu693Val)	15	Missense	Heterozygous	Reduced enzyme activity	DPC	1 Chinese	23
110	c.2132C>T (p.Pro711Leu)	15	Missense	Compound heterozygous with c.439G>A (p.Ala147Thr)	Inactivates protein	INAD	1 British & 1 American	1, 3
111	c.2215G>C (p.Asp739His)	16	Missense	Compound heterozygous with c.1061T>C (p.Leu354Pro)	Inactivates protein	ANAD	1 British & 1 American	1, 3
112	c.2218G>A (p.Gly740Arg)	16	Missense	Homozygous	Inactivates protein	ANAD	1 Saudi Arabian	25
113	c.2221C>T (p.Arg741Trp)	16	Missense	Homozygous, heterozygous or compound heterozygous with c.1770C>T (p.Asp590Glu) or c.1912G>A (p.Gly638Arg)	Inactivates protein	INAD	2 British, 2 American, 1 Pakistani, 1 Algerian & 1 Indian	1, 3, 9, 30, Present study
114	c.2222G>A (p.Arg741Gln)	16	Missense	Homozygous	Inactivates protein	DPC	1 Indian	5
115	c.2233C>T (p.Arg745Trp)	16	Missense	Compound heterozygous with c.1744G>T (p.Val582Leu)	Inactivates protein	INAD & ANAD	1 British & 1 American	1, 3
116	c.2234G>C (p.Arg745Pro)	16	Missense	Homozygous	Inactivates protein	INAD	2 Tunisian	30
117	c.2239C>T (p.Arg747Trp)	16	Missense	Homozygous	Inactivates protein	DPC	1 Pakistani	5
118	c.2251G>A	16	Missense	Homozygous	Inactivates	INAD	2 American	1, 3

	(p.Glu751Lys)				protein		& 1 British	
119	c.2251G>T (p.Glu751*)	16	Nonsense	Compound heterozygous with c.1957 G>A (p.Gly635Ser)	Premature protein truncation	INAD	1 Chinese	24
120	c.2261G>T (p.Gly754Val)	16	Missense	Compound heterozygous with c.372delC (p.His124Leufs*324)	Premature protein truncation	ANAD	1 Chinese	24
121	c.2339A>G (p.Asn780Ser)	17	Missense	Heterozygous	Inactivates protein	DPC	1 German	17
122	c.2341G>A (p.Ala781Thr)	17	Missense	Heterozygous	Inactivates protein	DPC	1 German	17
123	IVS16+1G>A	IVS 16	Splice site	Homozygous	Aberrant splicing	INAD	1 British, 1 Italian & 1 American	1, 3, 7
124	c.2491_2500del (p.Glu786Serfs*29)	17	Deletion	Homozygous	Premature protein truncation	INAD	1 Tunisian	30
125	c.2370T>G (p.Tyr790*)	17	Nonsense	Homozygous, heterozygous or compound heterozygous with c.691G>C (p.Gly231Arg) or c.1442T>A (p.Leu481Gln)	Premature protein truncation	INAD, ANAD & DPC	4 Caucasian of western European descent, 4 American, 1 British, 1 Portuguese & 1 Caucasian	1, 3, 8, 21, 28
126	c.2370_2371delTG (p.Tyr790*)	17	Deletion	Homozygous or compound heterozygous with c.238G>A (p.Ala80Thr), IVS5 -1G>T or c.1538_1541delACTG (p.Asp513fs*543	Premature protein truncation	INAD & ANAD	2 British & 3 American	1, 3
127	c.2375A>C (p.His792Pro)	17	Missense	Homozygous	Inactivates protein	INAD	1 Pakistani	28

128	c.2389C>T (p.Gln797*)	17	Nonsense	Heterozygous	Premature protein truncation	INAD	1 Chinese	24
129	c.2417C>G (p.Pro806Arg)	17	Missense	Heterozygous	Inactivates protein	DPC	12 Japanese, 1 Singaporean & 1 Taiwanese	13, 19, 20
